# Anaphylactic death due to bee sting – a comparison case study

**DOI:** 10.1186/1710-1492-10-S1-A21

**Published:** 2014-03-03

**Authors:** Lindsay Douglas, Shahin Zanganeh

**Affiliations:** 1 Schulich School of Medicine and Dentistry – Western University, Windsor, Ontario, Canada; 2 Windsor Allergy Asthma Associates, Windsor, Ontario, Canada

## Introduction

In 2000 the World Health Organization reported that in the USA there were 54 deaths attributed to bee stings from a population of 281 million. Only 0.4% of the population is allergic to stings. First line treatment of stings is Epinephrine best delivered when anaphylactic symptoms are first noted. In the spring of 2012 two males were stung in Windsor Essex County resulting cardiac arrest.

## Methods

We describe a comparison case study of two patients.

Patient A: A 45 Year-old male was stung by a wasp as he was putting on his shirt. Patient complained of chest pain and soon collapsed. Past medical history is unremarkable.

Patient B: A 48-Year old male stung multiple times while working outside. Patient became light-headed and passed out. Past history is significant for a venom allergy and left eye injury as a child.

## Results

Patient A: EMS notified, epinephrine administered with delay. Patient transferred to local hospital pulseless on arrival. He was resuscitated over a period of 45 minutes where he developed a poor pulse. Total IgE and specific IgE revealed an IgE of 149kU/L, Common Wasp 1.50 kU/L, Honey Bee 0.55 kU/L, Paper Wasp 2.83 kU/L, White-Faced Hornet 1.08 kU/L and Yellow Hornet 0.57 kU/L. Nine days after sting patient had worsening neurological status. A repeat CT of the head revealed a new left thalamic intracranial hemorrhage with mass effect. The following day support was withdrawn and the patient expired shortly after.

Patient B: EMS notified, epinephrine administered. Patient transferred to local hospital PEA on arrival. He was resuscitated and transferred to ICU. Specific IgE revealed Common Wasp 0.70 kU/L, Honey Bee <0.35 kU/L, Paper Wasp 0.35 kU/L, White-Faced Hornet 0.35 kU/L and Yellow Hornet <0.35 kU/L. Patient discharged home 11 days after sting. Patient noted to be neurologically stable with some short-term memory loss.

## Conclusion

Early recognition and treatment is crucial to patient survival with venom allergy. People at risk of insect sting anaphylaxis should be educated regarding measures to avoid insect stings. It is advised that an epinephrine auto injector be immediately available and administered as soon as a severe reaction is suspected.

